# Crystal structure of 2-{[1-(4-bromo­benz­yl)-1*H*-1,2,3-triazol-4-yl]meth­oxy}naph­thalene-1,4-dione

**DOI:** 10.1107/S2056989015004429

**Published:** 2015-03-11

**Authors:** Rajamani Raja, Subramani Kandhasamy, Paramasivam T. Perumal, A. SubbiahPandi

**Affiliations:** a Department of Physics, Presidency College (Autonomous), Chennai 600 005, India; b Organic Chemistry Division, CSIR Central Leather Research Institute, Chennai 600 020, India

**Keywords:** crystal structure, triazole, naphthalene, hydrogen bonds

## Abstract

In the title compound, C_20_H_14_BrN_3_O_3_, the benzene ring makes dihedral angles of 71.30 (11) and 68.95 (14)° with the naphthalene ring system and the triazole ring, respectively. The latter two ring systems are coplanar, with a dihedral angle of 2.92 (12)°. The O atoms deviate from the naphthalene ring system by 0.029 (2) and −0.051 (2) Å. In the crystal, mol­ecules are linked by C—H⋯O and C—H⋯N hydrogen bonds, forming ribbons parallel to (10-1). The ribbons are linked *via* C—H⋯O and π–π stacking inter­actions [centroid–centroid distance = 3.4451 (14) Å], forming slabs parallel to the *bc* plane.

## Related literature   

For some general background and examples of the pharmacological and biological activity of triazole and its derivatives, see, for example: Abu-Orabi *et al.* (1989 ▸); Demirbaş *et al.* (2002 ▸); Kritsanida *et al.* (2002 ▸). For the biological activity of naphthalene compounds, see, for example: Upadhayaya *et al.* (2010 ▸); Rokade & Sayyed (2009 ▸).
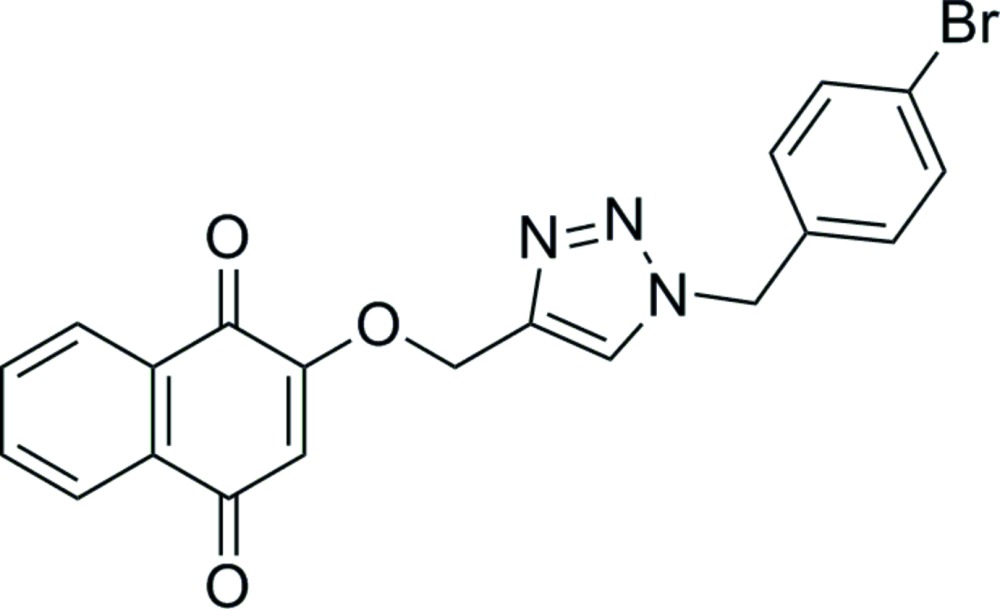


## Experimental   

### Crystal data  

C_20_H_14_BrN_3_O_3_
*M* *_r_* = 424.25
Monoclinic, 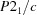

*a* = 16.4383 (5) Å
*b* = 13.1684 (4) Å
*c* = 8.2255 (2) Å
β = 90.827 (1)°
*V* = 1780.36 (9) Å^3^
*Z* = 4
Mo *K*α radiation
μ = 2.34 mm^−1^
*T* = 293 K
0.25 × 0.20 × 0.20 mm

### Data collection  

Bruker SMART APEXII CCD diffractometer
Absorption correction: multi-scan (*SADABS*; Bruker, 2008 ▸) *T*_min_ = 0.593, *T*_max_ = 0.652
17010 measured reflections
4415 independent reflections
2887 reflections with *I* > 2σ(*I*)
*R*_int_ = 0.031

### Refinement  

*R*[*F* ^2^ > 2σ(*F* ^2^)] = 0.044
*wR*(*F* ^2^) = 0.119
*S* = 1.01
4415 reflections
244 parameters
H-atom parameters constrained
Δρ_max_ = 0.42 e Å^−3^
Δρ_min_ = −0.93 e Å^−3^

### 

Data collection: *APEX2* (Bruker, 2008 ▸); cell refinement: *SAINT* (Bruker, 2008 ▸); data reduction: *SAINT*; program(s) used to solve structure: *SHELXS97* (Sheldrick, 2008 ▸); program(s) used to refine structure: *SHELXL97* (Sheldrick, 2008 ▸); molecular graphics: *PLATON* (Spek, 2009 ▸); software used to prepare material for publication: *SHELXL97* and *PLATON*.

## Supplementary Material

Crystal structure: contains datablock(s) global, I. DOI: 10.1107/S2056989015004429/su5090sup1.cif

Structure factors: contains datablock(s) I. DOI: 10.1107/S2056989015004429/su5090Isup2.hkl

Supporting information file. DOI: 10.1107/S2056989015004429/su5090Isup3.cml

**Fig. 1.**
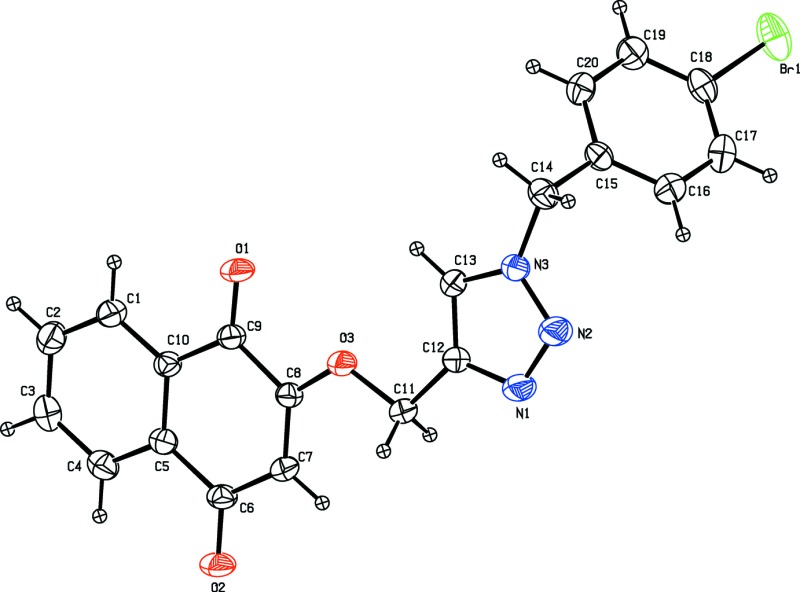
The molecular structure of the title compound, with atom labelling. Displacement ellipsoids are drawn at the 30% probability level.

**Fig. 2.**
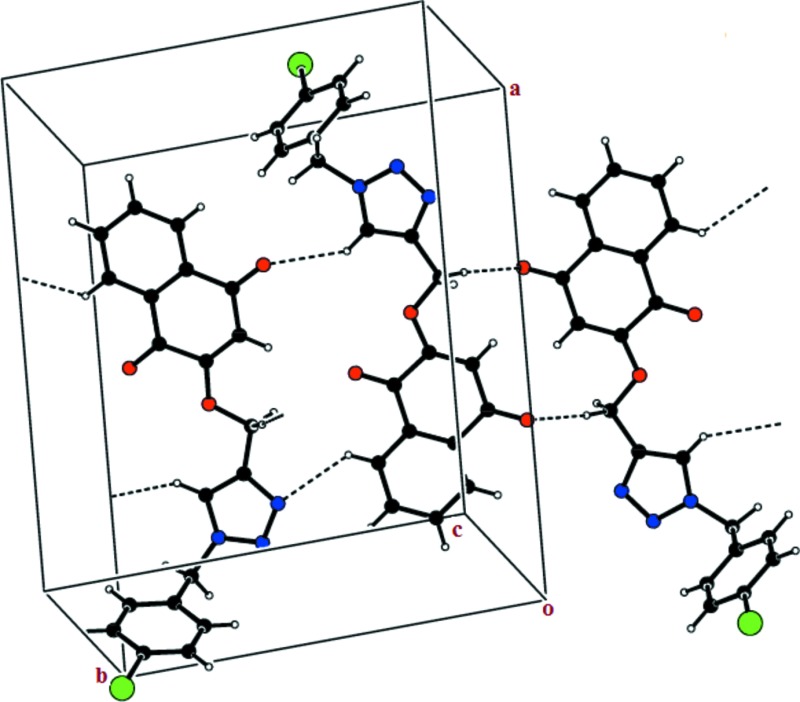
A partial view along the c axis of the crystal packing of the title compound. Hydrogen bonds are shown as dashed lines (see Table 1 for details).

**Fig. 3.**
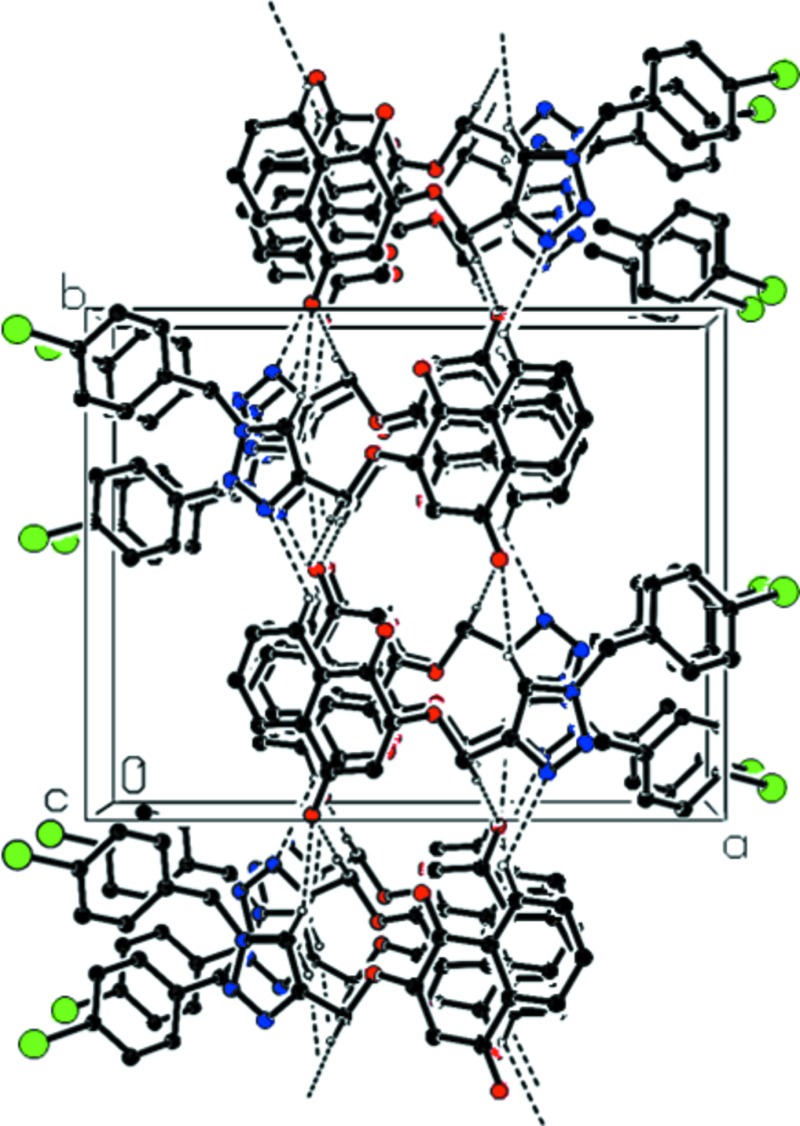
A perspective view along the c axis of the crystal packing of the title compound. Hydrogen bonds are shown as dashed lines (see Table 1 for details; H atoms not involved in these interactions have been omitted for clarity).

. DOI: 10.1107/S2056989015004429/su5090fig1.tif
The mol­ecular structure of the title compound, with atom labelling. Displacement ellipsoids are drawn at the 30% probability level.

c . DOI: 10.1107/S2056989015004429/su5090fig2.tif
A partial view along the *c* axis of the crystal packing of the title compound. Hydrogen bonds are shown as dashed lines (see Table 1 for details).

c . DOI: 10.1107/S2056989015004429/su5090fig3.tif
A perspective view along the *c* axis of the crystal packing of the title compound. Hydrogen bonds are shown as dashed lines (see Table 1 for details; H atoms not involved in these inter­actions have been omitted for clarity).

CCDC reference: 1052111

Additional supporting information:  crystallographic information; 3D view; checkCIF report

## Figures and Tables

**Table 1:** Hydrogen-bond geometry (, )

*D*H*A*	*D*H	H*A*	*D* *A*	*D*H*A*
C1H1N1^i^	0.93	2.57	3.251(3)	131
C13H13O2^i^	0.93	2.53	3.277(3)	138
C11H11*A*O2^ii^	0.97	2.46	3.425(3)	175

Symmetry codes: (i) 
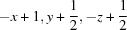
; (ii) 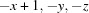.

## supplementary crystallographic information

### S1. Comment

Triazoles and triazole derivatives play an important role in pharmaceuticals, agrochemicals, dyes, photographic materials, and in corrosion inhibition and have many biological applications (Abu-Orabi *et al.*, 1989; Demirbaş *et al.*, 2002; Kritsanida *et al.*, 2002. Naphthalene derivatives has been identified as new range of potent antimicrobials effective against wide range of human pathogens and have diverse and interesting antibiotic properties with minimum toxicity (Rokade & Sayyed, 2009; Upadhayaya *et al.* 2010).

The molecular structure of the title compound is shown in Fig. 1. The benzene ring (C15-C20) makes dihedral angles of 71.30 (11) and 68.95 (14) ° with the naphthalene ring system and the triazole ring, respectively. The latter two rings are coplanar with a dihedral angle of 2.92 (12) °. Atoms O1 and O2 deviate from the naphthalene ring by 0.029 (2) and -0.051 (2) Å, respectively. Atom Br1 deviates from the benzene ring to which it is attached by -0.028 (1) Å

In the crystal, molecules are linked by C-H···O and C-H···N hydrogen bonds forming ribbons parallel to (101); see Table 1 and Fig. 2. The ribbons are linked by C-H···O hydrogen bonds and π–π stacking interactions [Cg2···Cg3^i^ = 3.4451 (14) Å; Cg2 and Cg3 are the centroids of rings naphthalene rings C1-C5/C10 and C5-C10, respectively; symmetry code: (i) x, -y+1/2, z-1/2], forming slabs parallel to the bc plane (Table 1 and Fig. 3).

### S2. Experimental

The triazole appended lawsone was synthesized in a two step procedure. To a solution of lawsone (0.87 g, 5 mmol) in DMF (20 ml) was added potassium carbonate (1.04 g, 7.5 mmol) and the solution was stirred at room temperature. Propargyl bromide (0.7 mL, 7.5 mmol) was added drop wise and the resulting mixture was allowed to stir overnight. After completion of the reaction, in the mixture was partitioned between DCM and water, and the DCM layer was collected. The aqueous layer was extracted three times with DCM. The combined organic extracts were dried over anhydrous Na_2_SO_4_, and concentrated under vacuum to obtain the desired propargyllated lawsone that was later converted to a triazole using click chemistry. Propargyllated lawsone (0.636 g, 3 mmol) was dissolved in 1:1 THF / H_2_O mixture and triethyl amine (0.7 ml, 5 mmol), sodium azide (0.26 g, 4 mmol), 4-bromobenzyl bromide (0.68 mL, 4 mmol) and cuprous iodide (catalytic amount) were added to this solution. The resulting mixture was allowed to stir overnight at room temperature. Upon completion of the reaction, the mixture was filtered, extract with ethyl acetate, concentrated under vacuum and then subjected to column chromatography to obtain the desired product. The overall yield was 0.61 g (60%). Colourless block-like crystals were obtained on slow evaporation of the solvent.

### S3. Refinement

The C-bound H atoms were positioned geometrically (C–H = 0.93–0.97 Å) and allowed to ride on their parent atoms with *U*_iso_(H) = 1.2*U*_eq_(C).

### Crystal data

**Table:**

C_20_H_14_BrN_3_O_3_	*F*(000) = 856
*M**_r_* = 424.25	*D*_x_ = 1.583 Mg m^−^^3^
Monoclinic, *P*2_1_/*c*	Mo *K*α radiation, λ = 0.71073 Å
Hall symbol: -P 2ybc	Cell parameters from 2887 reflections
*a* = 16.4383 (5) Å	θ = 1.2–28.3°
*b* = 13.1684 (4) Å	µ = 2.34 mm^−^^1^
*c* = 8.2255 (2) Å	*T* = 293 K
β = 90.827 (1)°	Block, colourless
*V* = 1780.36 (9) Å^3^	0.25 × 0.20 × 0.20 mm
*Z* = 4	

### Data collection

**Table:**

Bruker SMART APEXII CCD diffractometer	4415 independent reflections
Radiation source: fine-focus sealed tube	2887 reflections with *I* > 2σ(*I*)
Graphite monochromator	*R*_int_ = 0.031
ω and φ scans	θ_max_ = 28.3°, θ_min_ = 1.2°
Absorption correction: multi-scan (*SADABS*; Bruker, 2008)	*h* = −21→20
*T*_min_ = 0.593, *T*_max_ = 0.652	*k* = −16→17
17010 measured reflections	*l* = −10→10

### Refinement

**Table:**

Refinement on *F*^2^	Primary atom site location: structure-invariant direct methods
Least-squares matrix: full	Secondary atom site location: difference Fourier map
*R*[*F*^2^ > 2σ(*F*^2^)] = 0.044	Hydrogen site location: inferred from neighbouring sites
*wR*(*F*^2^) = 0.119	H-atom parameters constrained
*S* = 1.01	*w* = 1/[σ^2^(*F*_o_^2^) + (0.0595*P*)^2^ + 0.5827*P*] where *P* = (*F*_o_^2^ + 2*F*_c_^2^)/3
4415 reflections	(Δ/σ)_max_ = 0.003
244 parameters	Δρ_max_ = 0.42 e Å^−^^3^
0 restraints	Δρ_min_ = −0.93 e Å^−^^3^

### Special details

**Table:**

Geometry. All e.s.d.'s (except the e.s.d. in the dihedral angle between two l.s. planes) are estimated using the full covariance matrix. The cell e.s.d.'s are taken into account individually in the estimation of e.s.d.'s in distances, angles and torsion angles; correlations between e.s.d.'s in cell parameters are only used when they are defined by crystal symmetry. An approximate (isotropic) treatment of cell e.s.d.'s is used for estimating e.s.d.'s involving l.s. planes.
Refinement. Refinement of *F*^2^ against ALL reflections. The weighted *R*-factor *wR* and goodness of fit *S* are based on *F*^2^, conventional *R*-factors *R* are based on *F*, with *F* set to zero for negative *F*^2^. The threshold expression of *F*^2^ > σ(*F*^2^) is used only for calculating *R*-factors(gt) *etc*. and is not relevant to the choice of reflections for refinement. *R*-factors based on *F*^2^ are statistically about twice as large as those based on *F*, and *R*- factors based on ALL data will be even larger.

### Fractional atomic coordinates and isotropic or equivalent isotropic displacement parameters (Å^2^)

**Table:**

	*x*	*y*	*z*	*U*_iso_*/*U*_eq_	
C1	0.32817 (17)	0.37072 (18)	−0.0249 (3)	0.0477 (6)	
H1	0.3479	0.4340	0.0067	0.057*	
C2	0.25884 (18)	0.3640 (2)	−0.1206 (3)	0.0541 (7)	
H2	0.2316	0.4228	−0.1523	0.065*	
C3	0.22993 (16)	0.2711 (2)	−0.1692 (3)	0.0545 (7)	
H3	0.1838	0.2673	−0.2356	0.065*	
C4	0.26908 (16)	0.1828 (2)	−0.1200 (3)	0.0487 (6)	
H4	0.2488	0.1200	−0.1526	0.058*	
C5	0.33842 (14)	0.18813 (17)	−0.0221 (3)	0.0387 (5)	
C6	0.37999 (16)	0.09419 (17)	0.0346 (3)	0.0421 (6)	
C7	0.45299 (16)	0.10389 (17)	0.1362 (3)	0.0421 (6)	
H7	0.4800	0.0455	0.1704	0.051*	
C8	0.48218 (15)	0.19489 (17)	0.1817 (3)	0.0389 (5)	
C9	0.44255 (15)	0.29198 (17)	0.1280 (3)	0.0410 (5)	
C10	0.36854 (15)	0.28351 (16)	0.0242 (3)	0.0385 (5)	
C11	0.59314 (15)	0.12805 (17)	0.3357 (3)	0.0447 (6)	
H11A	0.6115	0.0873	0.2450	0.054*	
H11B	0.5606	0.0856	0.4060	0.054*	
C12	0.66411 (15)	0.17104 (17)	0.4275 (3)	0.0415 (5)	
C13	0.68666 (15)	0.26868 (17)	0.4532 (3)	0.0410 (5)	
H13	0.6606	0.3271	0.4158	0.049*	
C14	0.80758 (16)	0.3445 (2)	0.6053 (3)	0.0488 (6)	
H14A	0.8309	0.3247	0.7095	0.059*	
H14B	0.7750	0.4050	0.6222	0.059*	
C15	0.87538 (15)	0.36917 (18)	0.4899 (3)	0.0420 (5)	
C16	0.93614 (18)	0.2990 (2)	0.4612 (4)	0.0565 (7)	
H16	0.9345	0.2359	0.5116	0.068*	
C17	0.99932 (18)	0.3220 (2)	0.3580 (4)	0.0628 (8)	
H17	1.0402	0.2747	0.3394	0.075*	
C18	1.00118 (16)	0.4152 (2)	0.2834 (3)	0.0506 (6)	
C19	0.94163 (17)	0.4853 (2)	0.3095 (3)	0.0532 (6)	
H19	0.9433	0.5482	0.2583	0.064*	
C20	0.87863 (17)	0.4614 (2)	0.4131 (3)	0.0494 (6)	
H20	0.8378	0.5089	0.4308	0.059*	
N1	0.71901 (16)	0.11000 (16)	0.5018 (3)	0.0625 (7)	
N2	0.77460 (15)	0.16630 (17)	0.5737 (3)	0.0628 (7)	
N3	0.75486 (12)	0.26276 (15)	0.5446 (2)	0.0428 (5)	
O1	0.47077 (12)	0.37334 (12)	0.1695 (2)	0.0578 (5)	
O2	0.35311 (12)	0.01052 (13)	−0.0017 (2)	0.0591 (5)	
O3	0.54657 (11)	0.21317 (12)	0.2785 (2)	0.0480 (4)	
Br1	1.08857 (2)	0.44786 (3)	0.14393 (4)	0.07837 (17)	

### Atomic displacement parameters (Å^2^)

**Table:**

	*U*^11^	*U*^22^	*U*^33^	*U*^12^	*U*^13^	*U*^23^
C1	0.0497 (16)	0.0378 (13)	0.0558 (14)	0.0009 (12)	0.0050 (12)	−0.0021 (11)
C2	0.0514 (17)	0.0522 (16)	0.0586 (15)	0.0098 (13)	0.0028 (13)	0.0060 (12)
C3	0.0387 (15)	0.0711 (19)	0.0536 (14)	0.0025 (13)	0.0001 (12)	0.0037 (13)
C4	0.0466 (15)	0.0509 (15)	0.0486 (13)	−0.0089 (12)	0.0031 (11)	−0.0067 (11)
C5	0.0409 (13)	0.0379 (12)	0.0375 (11)	−0.0041 (10)	0.0084 (9)	−0.0056 (9)
C6	0.0474 (15)	0.0329 (12)	0.0461 (12)	−0.0055 (11)	0.0056 (11)	−0.0087 (10)
C7	0.0456 (15)	0.0298 (12)	0.0509 (13)	0.0017 (10)	−0.0001 (11)	−0.0048 (10)
C8	0.0381 (13)	0.0330 (12)	0.0458 (12)	−0.0029 (10)	0.0040 (10)	−0.0041 (9)
C9	0.0433 (14)	0.0319 (12)	0.0479 (12)	−0.0024 (10)	0.0071 (10)	−0.0026 (10)
C10	0.0407 (13)	0.0331 (12)	0.0420 (11)	−0.0008 (10)	0.0084 (10)	−0.0020 (9)
C11	0.0432 (14)	0.0306 (12)	0.0602 (14)	0.0006 (10)	−0.0015 (11)	−0.0038 (10)
C12	0.0398 (13)	0.0341 (12)	0.0508 (13)	−0.0019 (11)	0.0037 (10)	−0.0003 (10)
C13	0.0390 (13)	0.0358 (12)	0.0482 (12)	0.0036 (10)	−0.0021 (10)	0.0001 (10)
C14	0.0443 (15)	0.0543 (15)	0.0478 (13)	−0.0065 (12)	−0.0041 (11)	−0.0056 (11)
C15	0.0376 (13)	0.0474 (14)	0.0406 (11)	−0.0050 (11)	−0.0091 (10)	−0.0040 (10)
C16	0.0542 (17)	0.0477 (15)	0.0677 (17)	0.0023 (13)	0.0016 (13)	0.0069 (13)
C17	0.0464 (17)	0.0701 (19)	0.0719 (18)	0.0147 (15)	0.0026 (14)	−0.0014 (16)
C18	0.0389 (15)	0.0725 (18)	0.0403 (12)	−0.0077 (13)	−0.0046 (11)	−0.0019 (12)
C19	0.0519 (17)	0.0582 (16)	0.0493 (13)	−0.0001 (14)	−0.0044 (12)	0.0105 (12)
C20	0.0473 (16)	0.0517 (15)	0.0492 (13)	0.0077 (12)	−0.0039 (12)	0.0016 (11)
N1	0.0598 (16)	0.0377 (12)	0.0895 (17)	−0.0032 (11)	−0.0182 (13)	0.0130 (12)
N2	0.0595 (15)	0.0447 (13)	0.0836 (16)	−0.0032 (12)	−0.0210 (13)	0.0151 (12)
N3	0.0412 (12)	0.0389 (11)	0.0481 (11)	−0.0035 (9)	−0.0021 (9)	0.0019 (9)
O1	0.0615 (13)	0.0288 (9)	0.0826 (13)	−0.0067 (8)	−0.0128 (10)	−0.0080 (8)
O2	0.0655 (13)	0.0337 (9)	0.0777 (13)	−0.0108 (9)	−0.0115 (10)	−0.0110 (9)
O3	0.0449 (10)	0.0330 (9)	0.0657 (11)	−0.0025 (7)	−0.0115 (8)	−0.0047 (8)
Br1	0.0513 (2)	0.1308 (4)	0.05317 (19)	−0.00707 (18)	0.00537 (14)	0.01042 (17)

### Geometric parameters (Å, º)

**Table:**

C1—C2	1.379 (4)	C11—H11B	0.9700
C1—C10	1.384 (3)	C12—N1	1.348 (3)
C1—H1	0.9300	C12—C13	1.354 (3)
C2—C3	1.369 (4)	C13—N3	1.343 (3)
C2—H2	0.9300	C13—H13	0.9300
C3—C4	1.386 (4)	C14—N3	1.466 (3)
C3—H3	0.9300	C14—C15	1.509 (3)
C4—C5	1.388 (3)	C14—H14A	0.9700
C4—H4	0.9300	C14—H14B	0.9700
C5—C10	1.401 (3)	C15—C20	1.371 (3)
C5—C6	1.485 (3)	C15—C16	1.383 (4)
C6—O2	1.223 (3)	C16—C17	1.384 (4)
C6—C7	1.458 (4)	C16—H16	0.9300
C7—C8	1.342 (3)	C17—C18	1.373 (4)
C7—H7	0.9300	C17—H17	0.9300
C8—O3	1.337 (3)	C18—C19	1.364 (4)
C8—C9	1.498 (3)	C18—Br1	1.901 (3)
C9—O1	1.215 (3)	C19—C20	1.387 (4)
C9—C10	1.480 (3)	C19—H19	0.9300
C11—O3	1.433 (3)	C20—H20	0.9300
C11—C12	1.492 (3)	N1—N2	1.311 (3)
C11—H11A	0.9700	N2—N3	1.332 (3)
			
C2—C1—C10	120.2 (2)	N1—C12—C13	108.4 (2)
C2—C1—H1	119.9	N1—C12—C11	121.1 (2)
C10—C1—H1	119.9	C13—C12—C11	130.5 (2)
C3—C2—C1	120.3 (3)	N3—C13—C12	104.9 (2)
C3—C2—H2	119.8	N3—C13—H13	127.6
C1—C2—H2	119.8	C12—C13—H13	127.6
C2—C3—C4	120.4 (3)	N3—C14—C15	112.5 (2)
C2—C3—H3	119.8	N3—C14—H14A	109.1
C4—C3—H3	119.8	C15—C14—H14A	109.1
C3—C4—C5	120.1 (2)	N3—C14—H14B	109.1
C3—C4—H4	120.0	C15—C14—H14B	109.1
C5—C4—H4	120.0	H14A—C14—H14B	107.8
C4—C5—C10	119.1 (2)	C20—C15—C16	118.7 (2)
C4—C5—C6	120.7 (2)	C20—C15—C14	120.9 (2)
C10—C5—C6	120.1 (2)	C16—C15—C14	120.4 (2)
O2—C6—C7	120.7 (2)	C15—C16—C17	120.6 (3)
O2—C6—C5	120.7 (2)	C15—C16—H16	119.7
C7—C6—C5	118.6 (2)	C17—C16—H16	119.7
C8—C7—C6	121.7 (2)	C18—C17—C16	119.4 (3)
C8—C7—H7	119.1	C18—C17—H17	120.3
C6—C7—H7	119.1	C16—C17—H17	120.3
O3—C8—C7	127.1 (2)	C19—C18—C17	120.9 (3)
O3—C8—C9	111.04 (19)	C19—C18—Br1	119.5 (2)
C7—C8—C9	121.8 (2)	C17—C18—Br1	119.6 (2)
O1—C9—C10	122.4 (2)	C18—C19—C20	119.2 (3)
O1—C9—C8	120.5 (2)	C18—C19—H19	120.4
C10—C9—C8	117.12 (19)	C20—C19—H19	120.4
C1—C10—C5	119.9 (2)	C15—C20—C19	121.2 (3)
C1—C10—C9	119.5 (2)	C15—C20—H20	119.4
C5—C10—C9	120.6 (2)	C19—C20—H20	119.4
O3—C11—C12	106.23 (18)	N2—N1—C12	108.9 (2)
O3—C11—H11A	110.5	N1—N2—N3	107.0 (2)
C12—C11—H11A	110.5	N2—N3—C13	110.8 (2)
O3—C11—H11B	110.5	N2—N3—C14	119.9 (2)
C12—C11—H11B	110.5	C13—N3—C14	129.3 (2)
H11A—C11—H11B	108.7	C8—O3—C11	117.99 (18)
			
C10—C1—C2—C3	−0.7 (4)	O3—C11—C12—C13	−2.2 (4)
C1—C2—C3—C4	1.3 (4)	N1—C12—C13—N3	−0.5 (3)
C2—C3—C4—C5	−0.6 (4)	C11—C12—C13—N3	179.7 (2)
C3—C4—C5—C10	−0.8 (4)	N3—C14—C15—C20	114.2 (3)
C3—C4—C5—C6	178.7 (2)	N3—C14—C15—C16	−66.6 (3)
C4—C5—C6—O2	−1.2 (4)	C20—C15—C16—C17	0.6 (4)
C10—C5—C6—O2	178.2 (2)	C14—C15—C16—C17	−178.7 (2)
C4—C5—C6—C7	179.5 (2)	C15—C16—C17—C18	−0.3 (4)
C10—C5—C6—C7	−1.1 (3)	C16—C17—C18—C19	−0.1 (4)
O2—C6—C7—C8	−177.9 (2)	C16—C17—C18—Br1	179.2 (2)
C5—C6—C7—C8	1.3 (4)	C17—C18—C19—C20	0.1 (4)
C6—C7—C8—O3	177.6 (2)	Br1—C18—C19—C20	−179.1 (2)
C6—C7—C8—C9	−1.3 (4)	C16—C15—C20—C19	−0.6 (4)
O3—C8—C9—O1	1.8 (3)	C14—C15—C20—C19	178.7 (2)
C7—C8—C9—O1	−179.2 (2)	C18—C19—C20—C15	0.2 (4)
O3—C8—C9—C10	−178.0 (2)	C13—C12—N1—N2	0.4 (3)
C7—C8—C9—C10	1.0 (3)	C11—C12—N1—N2	−179.8 (2)
C2—C1—C10—C5	−0.6 (4)	C12—N1—N2—N3	−0.1 (3)
C2—C1—C10—C9	−179.5 (2)	N1—N2—N3—C13	−0.3 (3)
C4—C5—C10—C1	1.4 (3)	N1—N2—N3—C14	−178.0 (2)
C6—C5—C10—C1	−178.1 (2)	C12—C13—N3—N2	0.5 (3)
C4—C5—C10—C9	−179.8 (2)	C12—C13—N3—C14	177.9 (2)
C6—C5—C10—C9	0.8 (3)	C15—C14—N3—N2	86.2 (3)
O1—C9—C10—C1	−1.7 (4)	C15—C14—N3—C13	−91.0 (3)
C8—C9—C10—C1	178.1 (2)	C7—C8—O3—C11	3.3 (4)
O1—C9—C10—C5	179.4 (2)	C9—C8—O3—C11	−177.8 (2)
C8—C9—C10—C5	−0.8 (3)	C12—C11—O3—C8	175.2 (2)
O3—C11—C12—N1	178.0 (2)		

### Hydrogen-bond geometry (Å, º)

**Table:**

*D*—H···*A*	*D*—H	H···*A*	*D*···*A*	*D*—H···*A*
C1—H1···N1^i^	0.93	2.57	3.251 (3)	131
C13—H13···O2^i^	0.93	2.53	3.277 (3)	138
C11—H11*A*···O2^ii^	0.97	2.46	3.425 (3)	175

Symmetry codes: (i) −*x*+1, *y*+1/2, −*z*+1/2; (ii) −*x*+1, −*y*, −*z*.
