# Supplementary figures and images for: Crystal structure of 2-{[1-(4-bromo­benz­yl)-1H-1,2,3-triazol-4-yl]meth­oxy}naph­thalene-1,4-dione
Source: Acta Crystallogr E Crystallogr Commun. 2015 Mar 11;71(Pt 4):o231–2. doi: 10.1107/S2056989015004429 (PMC4438838; doi:10.1107/S2056989015004429)

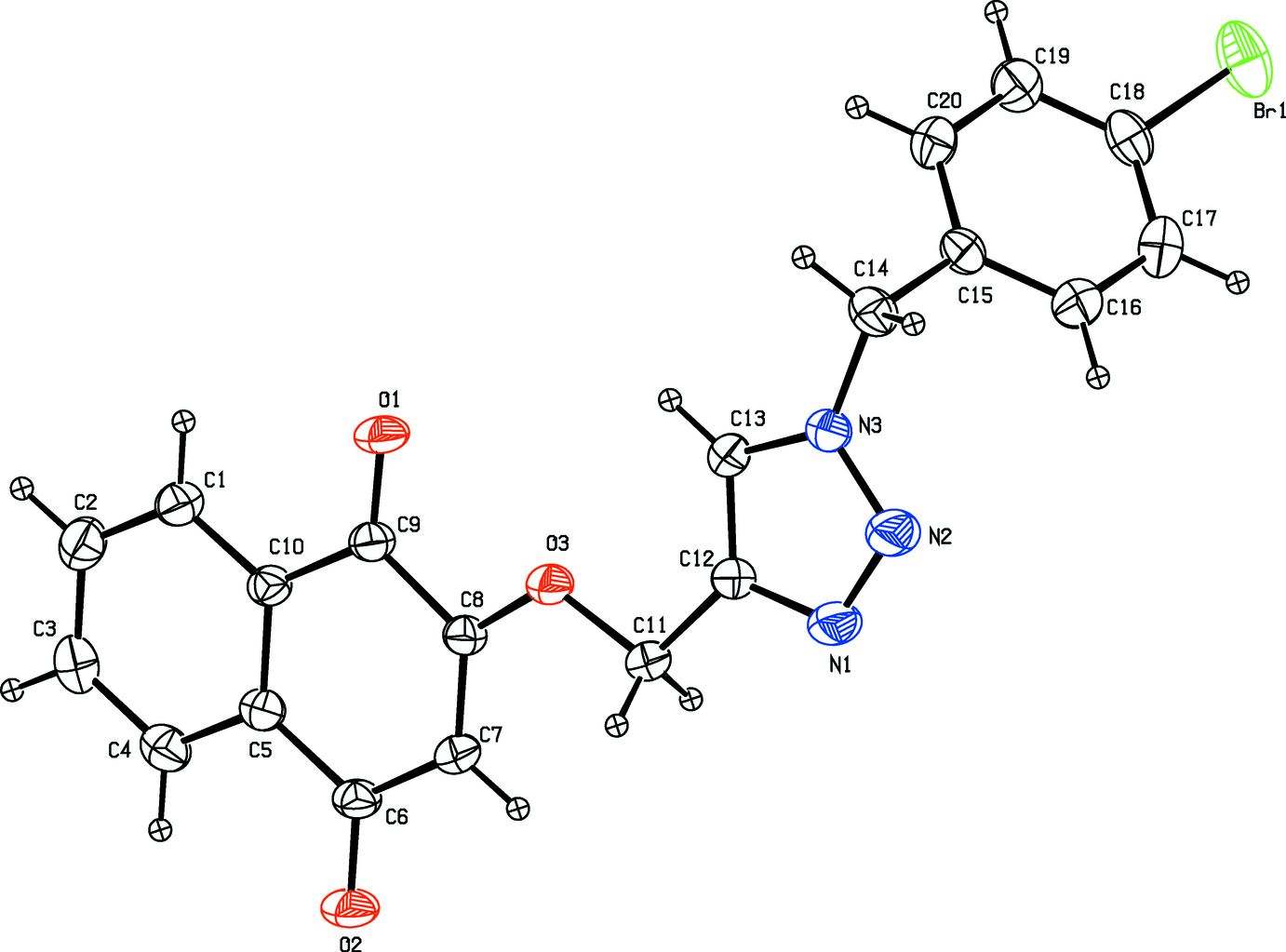

Supplement: Supplementary file 4 [file e-71-0o231-fig1.tif]

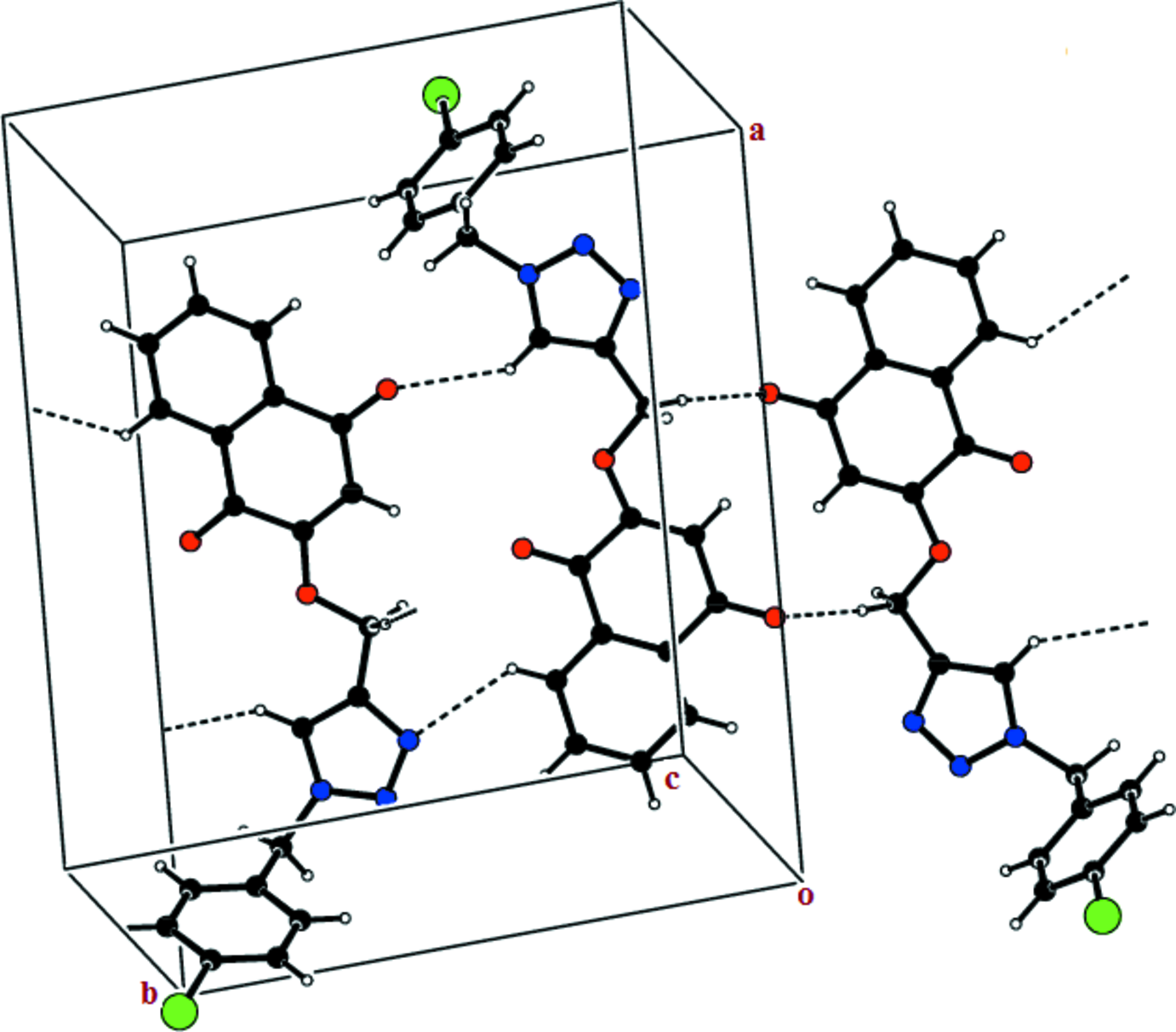

Supplement: Supplementary file 5 [file e-71-0o231-fig2.tif]

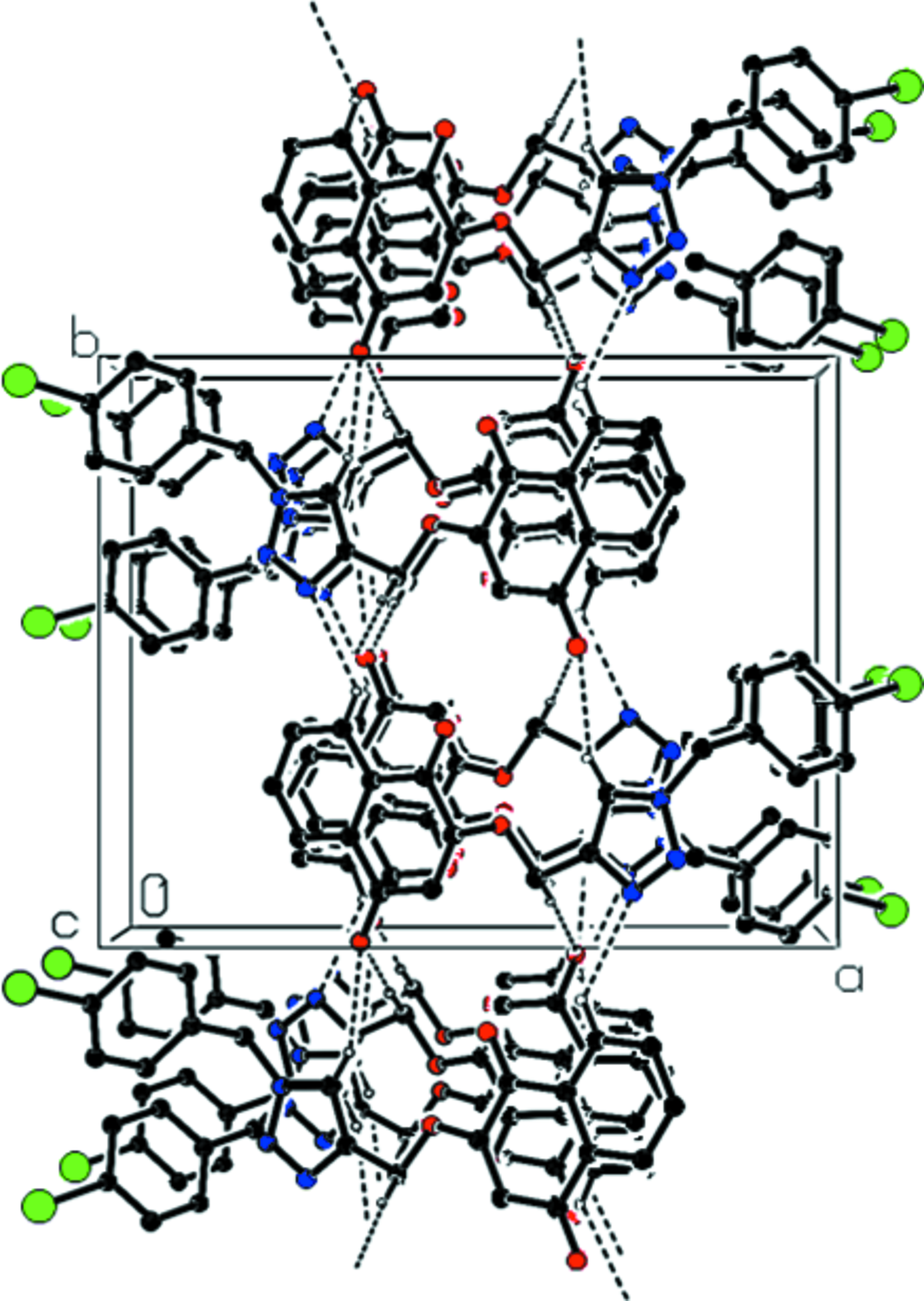

Supplement: Supplementary file 6 [file e-71-0o231-fig3.tif]
